# Factors Associated with Mental and Behavioral Health Programming in U.S. Rural, Urban and Suburban Congregations

**DOI:** 10.1007/s10943-025-02403-6

**Published:** 2025-08-09

**Authors:** CeRon Ford, Katie Rydberg, Carrie Henning-Smith

**Affiliations:** Division of Health Policy and Management, University of Minnesota School of Public Health, Mayo Mail Code 729, 420 Delaware Street S.E., Minneapolis, MN 55455 USA

**Keywords:** Mental health programming, Behavioral health programming, Faith-based, Congregations, National Congregations Study

## Abstract

In the United States (U.S.), rural residents experience poorer mental health outcomes and more barriers to accessing mental health care than urban residents. Faith-based institutions provide mental and behavioral health programming that can fill gaps in accessibility to mental health care. This study’s primary objective was to examine specific factors associated with mental and behavioral health programming in rural, urban and suburban congregations. Data come from 1,262 U.S. congregations in the 2018–2019 National Congregations Study. We conducted logistic regression models to analyze the relationship between mental health programming and support and county classification, while controlling for sociodemographic and sociopolitical factors. We found that urban and suburban congregations with more than 500 attendees were more likely than smaller congregations to provide mental and behavioral health programming and support (OR = 3.43, *p* < 0.001). Urban and suburban congregations that made an effort to get people registered to vote (OR = 5.36, *p* < 0.01) and discussed science and religion (OR = 3.16, *p* < 0.05) were more likely to provide programming and support. Rural congregations (OR = 4.37, *p* < 0.05) and urban and suburban congregations (OR = 2.61, *p* < 0.05) that followed the prosperity gospel were also more likely to provide programming and support. While urban and suburban congregations are more likely to provide mental health programming and support than rural congregations, several complex sociodemographic, sociopolitical, and health-related characteristics impact the likelihood of providing mental health programs. Further research is needed to examine the quality and utilization of congregational mental health programming and/or support by rural, suburban and urban residents.

**Supplementary Information:**

The online version contains supplementary material available at 10.1007/s10943-025-02403-6.

## Introduction

Mental health outcomes across the United States (U.S.) are poor, and outcomes are even worse in rural areas (Rural Health Information Hub, 2024). Rural residents experience higher rates of depression, anxiety, and suicide than urban residents (Morales et al., 2020; Steelesmith et al., 2019), as well as higher rates of substance use (Friesen et al., 2022; Lister et al., 2020). Generally, rural residents suffer from poorer health compared to urban residents (García et al., 2024), which may compound their mental health challenges. There are many contributors to rural mental health inequities, but a key contributor is limited access to mental health care (Palomin et al., 2023; Trawver et al., 2020).

### ***Barriers to Accessing Mental Health Care in Rural Areas***

Over two-thirds of rural areas are considered Health Professional Shortage Areas, presenting a challenge for rural residents to access mental health care which can negatively impact their mental health outcomes (Health Resources & Services Administration, 2024; Morales et al., 2020; Palomin et al., 2023; Trawver et al., 2020). Rural mental health care workforce shortages are large and persistent (Henning-Smith et al., 2024) due to various factors impacting providers’ decisions to practice in rural communities. Rural residents have lower rates of health insurance and fewer financial resources to afford mental health care out of pocket (Day, 2019). Compared to urban areas, rural areas have limited access to specialists (Shaw, 2024), and declining access to many types of healthcare facilities, including hospitals (Mullens et al., 2024), pharmacies (Adepoju et al., 2023), and nursing homes (Sharma et al., 2021).

### Rural Strengths and Diversity

Rural areas have many strengths, including strong social cohesion (Avery et al., 2021; Jones et al., 2012) and stronger ties to faith-based institutions than urban areas (Hatcher, 2018). Faith-based institutions often fill gaps in people’s lives where other services are not available, affordable, or welcoming, including social services, transportation, food access, emergency financial assistance (Chaves, 1999), and health services or health programs (Trinitapoli et al., 2009). Research suggests that faith-based institutions and religious congregations provide a more welcoming and comfortable environment for rural residents seeking mental health and substance use support, particularly for those who experience stigma or shame around seeking care through traditional community settings (Baldwin & Poje, 2020; Grim & Grim, 2019; Leavey et al., 2012; Torres et al., 2023; Wang et al., 2003; White et al., 2012; Wong et al., 2018a, 2018b). Other studies argue relying on faith-based institutions and religious congregations for mental health and substance use issues are potentially harmful to congregants due to stigmatizing encounters with religious leaders (Holleman & Chaves, 2023; Karadzhov & White, 2020; Payne, 2008).

Rural areas are not monolithic and can vary based on race, ethnicity, educational attainment, and other factors (Collinsworth, 2020; Palomin et al., 2023). Access to care and mental and behavioral health outcomes varies by sociodemographic factors (Baldwin & Poje, 2020; Wong et al., 2018a, 2018b; Wong et al., 2018a, 2018b). Congregations differ in their theological underpinnings in ways that impact their involvement with and approach to mental and behavioral health (Webb, 2012). Such differences may also be borne out in congregations’ involvement with and approach to political and societal issues.

For example, a church teaching the “prosperity gospel” may place the responsibility for health and well-being on individual congregants, rather than addressing other structural issues (Webb, 2012). We can improve access to mental health care for rural residents by better understanding the culture, values, and beliefs within rural communities (Jensen et al., 2020). This includes understanding how sociodemographic and sociopolitical characteristics influence the likelihood of congregations offering mental health programming.

### Factors Associated with Religious Congregations

Research has identified several factors associated with religious congregations that offer mental health and substance use programming and support. Several studies suggest that religious congregations with significantly more attendees and with significantly more high-income attendees are more likely to provide mental health programming (Holleman, 2023; Torres et al., 2023; Wong et al., 2018a, 2018b; Wong et al., 2018a, 2018b). Congregations with larger amounts of younger attendees and larger amounts of unemployed attendees are more likely to offer substance use programming (Torres et al., 2023).

Research using data from the National Congregations Study (NCS) shows that clergy characteristics—such as age (Wong et al., 2018a, 2018b), U.S. citizenship (Torres et al., 2023), and education level (Wong et al., 2018a, 2018b)—are linked to congregations’ provision of mental health programming. Race influences the provision of mental health programming, as congregations with larger amounts of Black populations are more likely to offer such programming (Holleman, 2023; Tsitsos, 2003; Wong et al., 2018a, 2018b).

### Purpose

Although some studies examine congregational mental health programming by geographical location, little is known about how related factors vary across rural, urban and suburban areas using the 2018–19 NCS data. For instance, Hidalgo et al. (2019) examined faith-based substance treatment programs that were implemented throughout urban congregations within Los Angeles County (Hidalgo et al., 2019). Research by Torres et al. (2022) used the 2012 NCS and discovered that congregational characteristics like urban vs. rural settings were not associated with providing programming for attendees with substance use issues. Recent efforts by Holleman (2023) examine mental illness and substance use programming provided by religious congregations, but do not assess variation in urban–rural county classification. Faith-based mental and behavioral health programming, particularly in rural areas, remains poorly understood; this study examines specific factors associated with such programming across rural, urban and suburban congregations.

## Methods

### Data Source

Data were obtained from the NCS, a national survey examining the characteristics and practices of U.S. congregations across denominations (Chaves et al., 2020). The NCS employs a probability sample of U.S. congregations aimed at understanding the religious landscape across religions. The sampling frame includes all known U.S. congregations, obtained from various sources such as denominational lists, religious directories, and other organizational databases.

Congregations are stratified by region, rurality, and religious tradition to ensure adequate representation across different geographic and denominational categories (Chaves et al., 2020). Congregational leaders (e.g., clergy, pastors, or other designated representatives) were identified and contacted to participate in the study, which occurred through mail, telephone, and web-based surveys (Chaves et al., 2020). The survey collects data on congregational demographics, religious beliefs and practices, social outreach programs, and organizational structure.

The 2018–2019 NCS Wave IV (NCS-IV) included 1,262 congregations and were collected between July 2018 and September 2019 with approximately 91% gathered through telephone and 9% through in-person interviews (Chaves et al., 2020). About 94% of interviews were conducted with staff from each congregation and about 6% of interviews were conducted with non-staff congregational leaders. Clergy were the most common respondents, accounting for 75% of interviews (Chaves et al., 2020).

The reported response rate for NCS-IV was 69%. The response rate was calculated using the conservative"RR3"method recommended by the American Association for Public Opinion Research and incorporates the 2018 General Social Survey’s 59% weighted response rate, which reduces the NCS-IV response rate to 49%. The NCS-IV cooperation rate, described as the percentage of contacted congregations who agreed to participate, was 74%.

## Measures

### Mental and Behavioral Health Programming and Support

We created a composite variable combining four measures to assess whether congregations offered any mental and behavioral health programming, including substance use support. Our composite variable includes four measures: (1) offered programs that involved mental health, (2) offered programs that involved substance abuse or addiction, (3) offered support for people with mental illness, and (4) offered support for people struggling with drug or alcohol abuse. Separately, these four measures were coded dichotomously—yes or no. We coded our composite variable dichotomously—"any programs/support” versus “no programs/support”—to indicate whether the congregation responded “yes” to offering any mental and behavioral health programming and support.

The survey included a measure of whether the congregation had “participated in or supported social service, community development, or neighborhood organizing projects” related to substance use. We did not include this measure in our analysis as it was unclear whether such efforts are provided by the congregation or are supported in another way. Prior research has shown that only a small percentage (< 3%) of congregations engaged in such activities (Holleman, 2023).

### Urban, Suburban and Rural County Classifications

NCS-IV data measures urban–rural classification as inside urbanized areas, inside urbanized clusters, and rural based on whether the congregation's census tract is predominantly urban, predominantly suburban, or predominantly rural. We defined this variable using the U.S. Census Bureau’s definitions: “Urban” indicates areas comprising 50,000 people or more; “Suburban” indicates areas comprising at least 2,500 and fewer than 50,000 people; and “Rural” encompasses all population, housing, and territory not included within an urban area (Chaves et al., 2020). We combined suburban congregations and urban congregations (*n* = 1,072) to compare with rural congregations (*n* = 190).

### Sociodemographic Characteristics

We provided sociodemographic characteristics about the lead clergy, the congregation, and the congregants. Clergy sociodemographic characteristics included the lead clergy’s race/ethnicity and sex. “Race and ethnicity” consist of six categories: White, Black or African American, Hispanic, Asian or Pacific Islander, Other, and unknown. The clergy’s sex was coded into three categories: male, female, and unknown. The congregant’s sociodemographic characteristics included: the percentage of female congregation attendees; percentage of attendees with a bachelor’s degree; congregation’s region, percentage of White, Black, Latino, Asian, and Other congregates; congregations that were 80% Black or African American; and congregations with at least 30% of attendees that were below the federal poverty level (FPL).

The percentage of female congregation attendees; the percentage of attendees with a bachelor’s degree; and the percentage of White, Black, Latino, Asian, and Other congregates were each coded into four categories: 0–25%, 25–49%, 50–74%, and 75–100%. The congregation’s region was coded into four categories: Northeastern, Midwestern, Southern, and Western. Congregations that were 80% Black or African American, and congregations with at least 30% of attendees that were below the FPL were coded dichotomously—yes or no. These characteristics were included to ascertain which congregational-level factors are associated with mental health programming.

Congregational sociodemographic characteristics included the following: number of adult attendees, official founding year, building ownership, building type used for worship, and total budget. The congregation’s number of adult attendees was dichotomized: less than 500 vs 500 or more and official founding year was coded into four categories: before 1900, 1900–1949, 1950–1999, and 2000–2018. Building ownership was dichotomized: belongs to the congregation or denomination vs belongs to another group and building type used for worship was coded into 4 categories: School, Storefront, Church/Synagogue/Temple/Mosque, and Other. Total budget for the most recent fiscal year was coded into five categories: less than $20 K; $20 K—$49,999; $50 K—$249,999; $250 K—$999,999; and greater than $1,000,000 +.

Additional congregational characteristics included were affiliation with a denomination convention or some similar kind of association, had a website on the Internet, had a Facebook page, and operated with a formal written annual budget. All of these responses were dichotomized—yes or no. We included a congregational characteristic that is sometimes referred to as the “prosperity Gospel” (Cornelio et al., 2020). Prosperity gospel was measured by the question, “Some religious groups teach that God gives financial wealth and good physical health to those with enough faith. Does your congregation teach this?”. Responses were dichotomized—yes or no.

### Sociopolitical Characteristics

We included measures of social and political factors that identify whether a congregation hosts groups, meetings, classes, or events to put mental health programming and support within a broader context of congregational activities. Sociopolitical characteristics included the following: discussed politics, an effort to get people registered to vote, discussed science and religion, discussed race relations, an effort to get people out to vote during an election, discussed race and police, and discussed issues related to sexual orientation and gender. All these sociopolitical characteristics were coded dichotomously—yes or no.

Other sociopolitical characteristics that we included consisted of the following: lobbied or marched in activities related to abortion; lobbied or marched in activities related to lesbian, gay, bisexual, or transgender (LGBT) people; and allowed same-sex weddings in the congregation’s building. We included a follow-up question to assess whether there had been lobbying or marching activity related to abortion, asking, “Was that on the pro-life side or the pro-choice side?”. Responses were coded into three categories: pro-life, pro-choice, and no answer. We included these characteristics to assess whether the provision of mental health programming is associated with political leanings and/or other cultural and political factors, although it is not always possible to tell which stance a congregation took on a particular issue.

### Health-related Programming Characteristics

Health-programming characteristics included information about whether a congregation offered either a health-related program, a health-related group, health-related support, or has hosted a specific health-related meeting. We coded the following responses dichotomously—yes or no—for congregations’ health-related programming and support characteristics: programs that involved health education, programs that focused on physical health needs, programs that targeted physical fitness, support to persons living with HIV/AIDS, and programs that exercised or promoted physical activity. We did not include these variables in the multiple logistic regression (MLR) models because they were closely correlated with our outcome variable. However, we included these in our descriptive analyses to provide a fuller picture of the involvement of rural and urban congregations in health-related programming.

### Analysis

We conducted two descriptive statistical analyses (DSA) and two MLR models. The first DSA summarized all of the sample’s characteristics across county classifications. The second DSA summarized sociodemographic and sociopolitical characteristics among congregations that provided mental and behavioral health programs and support. The first MLR model analyzes the relationship between select sociodemographic characteristics and county classification among congregations that provided mental health programming and support.

The second MLR model analyzes the relationship between select sociodemographic and sociopolitical characteristics and county classification among congregations that provided mental health programming and support. Results from the MLR models are presented as adjusted odds ratios (OR) and 95% confidence intervals (CI). All analyses were conducted separately for rural, urban and suburban areas in Stata version 18.0, using indicators for missing data to retain all observations.

## Results

### Sociodemographic, Sociopolitical, and Health-Related Characteristics

Approximately 25% of all congregations were in predominantly rural areas, and most rural congregations (62.8%) and urban and suburban congregations (41.0%, *p* < 0.01) were in the U.S. Southern region (see Supplemental Table 1 further analysis). Lead clergies were predominantly male in rural areas (80.4%) and urban and suburban areas (82.1%). Congregants were majority female, as 94.0% of rural congregations had > 50% female congregants and 92.3% of urban and suburban congregations had > 50% female congregants.

**Table 1:** Sociodemographic Factors and Mental and Behavioral Health Programming Among U.S. Congregations (N = 1,262)^a^

Sociodemographic characteristics	Urban and suburban (*N* = 1,072)	Rural (*N* = 190)
	OR (95% CI)	OR (95% CI)
*Clergy sex*		
Male	Ref	Ref
Female	1.54 (0.52–4.58)	0.29 (0.07–1.36)
*Number of adult congregants*		
Less than 500	Ref	Ref
Greater than 500	3.43 (1.69–6.99)***	0.16 (0.01–1.91)
*Percent of female congregants*		
Less than 50%	Ref	Ref
Greater than 50%	0.83 [0.22–3.12]	0.87 [0.09–8.84]
*Percentage of congregants with a bachelor’s degree*		
< 25%	Ref	Ref
25%−49%	0.76 (0.28–2.06)	1.88 (0.48–7.43)
50%−74%	0.94 (0.34–2.59)	3.13 (0.63–15.73)
75% +	0.77 (0.27–2.21)	1.05 (0.18–6.09)
*Year congregation was officially founded*		
Before 1900	Ref	Ref
1900–1949	0.58 (0.23–1.47)	0.60 (0.15–2.47)
1950–1999	0.64 (0.24–1.75)	3.85 (0.91–16.34)*
2000–2018	0.44 (0.15–1.33)	2.74 (0.09–85.21)
*Building ownership*		
Belongs to congregation or denomination	Ref	Ref
Belongs to another group	0.74 (0.23–2.38)	1.52 (0.16–14.19)
30% of congregation below federal poverty line^b^	0.90 (0.38–2.13)	0.74 (0.11–4.96)
Congregation affiliated with a denomination, convention, or other kind of association^b^	0.70 (0.24–1.98)	2.64 (0.44–15.97)
Congregation has a website on the internet^b^	1.31 (0.48–3.60)	1.34 (0.38–4.70)
Congregation has a Facebook page^b^	2.55 (0.94–6.87)*	0.94 (0.28–3.23)
Operates with a formal, written annual budget^b^	1.15 (0.41–3.21)	2.93 (0.65–13.31)
*Total budget for most recent fiscal year*		
Less than $250 K	Ref	Ref
Greater than $250 K	1.14 (0.53–2.45)	1.19 (0.20–7.21)
Follows the prosperity gospel^b^	2.61 (1.06–6.41)**	4.37 (1.06–18.03)**

*OR* odds ratio, *CI* confidence interval. ***p < 0.001; **p < 0.05; *p < 0.10

^a^Data are from the 2018‐2019 National Congregations Study

^b^0 = no, 1 = yes

Rural (99.4%) and urban and suburban congregations (94.9%, *p* < 0.01) were similar in terms of having fewer than 500 adult attendees. Rural congregations were less likely to have > 50% of attendees with a bachelor’s degree compared to urban and suburban congregations (17.9% vs. 44.5%, *p* < 0.01). Rural congregations were less likely to have 30% of their congregation below the FPL than urban and suburban congregations (8.3% vs. 17.6%, *p* < 0.05). Rural congregations were more likely to have > 75% of non-Hispanic white attendees than urban and suburban congregations (72.2% vs. 50.0%, *p* < 0.01).

Among sociopolitical characteristics, urban and suburban congregations were more likely to have held groups, meetings, classes, or events to discuss issues related to race and race relations than rural congregations (32.7% vs. 16.6%, *p* < 0.05). More urban and suburban congregations held groups, meetings, classes, or events to discuss issues related to sexual orientation or gender identity than rural congregations (14.6% vs. 8.5%, *p* < 0.05). Rural and urban/suburban congregations were similar in terms of allowing a wedding of two people of the same sex to take place in their building, discussing politics, discussing voting during an election, lobbying or marching activities related to abortion, and being pro-choice versus pro-life.

Among health-related characteristics, urban and suburban congregations were more likely to have provided health-focused programs involving physical fitness than rural congregations (1.2% vs. 0.1%, *p* < 0.05). The rate of urban and suburban congregations that offered support to persons living with HIV/AIDS was about 3 times more than rural congregations (16.6% vs. 5.2%, *p* < 0.05). More urban and suburban congregations offered support to promote exercise or physical activity than rural congregations (40.9% vs. 21.2%, *p* < 0.01).

### Mental and Behavioral Health Programming and Support

About 58.0% of rural congregations did not have programs and/or support for people with mental illness while 52% of urban and suburban congregations offered some sort of program and/or support (see Supplemental Table 2 for further analysis). More rural congregations did not have programs and/or support for people struggling with drug or alcohol abuse than urban and suburban congregations (64.0% vs. 54.4%). About 80.0% of rural congregations and about 72.0% of urban and suburban congregations did not have any programs for people with mental health conditions. None of the differences were statistically significant.

**Table 2:** Sociodemographic and Sociopolitical Characteristics Associated with Mental and Behavioral Health Programming Among U.S. Congregations (N = 1,262)^a^

Sociodemographic and Sociopolitical Characteristics	Urban and suburban (*N* = 1,072)	Rural (*N* = 190)
	OR (95% CI)	OR (95% CI)
*Sociodemographic Characteristics*		
Clergy sex		
Male	Ref	Ref
Female	1.23 (0.40–3.76)	0.22 (0.04–1.22)*
Number of adult congregants		
Less than 500	Ref	Ref
Greater than 500	3.36 (1.42–7.93)**	0.06 (0.00–1.75)*
Percent of female congregants		
Less than 50%	Ref	Ref
Greater than 50%	0.60 (0.15–2.43)	1.18 (0.14–10.20)
Percentage of congregants with a bachelor’s degree		
< 25%	Ref	Ref
25%−49%	0.91 (0.32–2.60)	1.37 (0.28–6.82)
50%−74%	1.48 (0.50–4.37)	2.92 (0.43–19.69)
75% +	0.82 (0.27–2.49)	0.48 (0.11–2.11)
Year congregation was officially founded		
Before 1900	Ref	Ref
1900–1949	0.49 (0.17–1.39)	0.32 (0.06–1.73)
1950–1999	0.50 (0.17–1.45)	2.60 (0.67–10.18)
2000–2018	0.26 (0.07–0.91)**	1.11 (0.08–15.38)
Building ownership		
Belongs to congregation or denomination	Ref	Ref
Belongs to another group	1.62 (0.50–5.28)	2.23 (0.30–16.38)
30% of congregation are below federal poverty line^b^	0.72 (0.25–2.05)	0.31 (0.03–3.76)
Congregation affiliated with a denomination, convention, or other kind of association^b^	0.52 (0.19–1.47)	3.65 (0.44–30.50)
Congregation has a website on the internet	0.93 (0.35–2.52)	2.78 (0.56–13.90)
Congregation has a Facebook page^b^	2.11 (0.77–5.82)	0.60 (0.14–2.60)
Operates with a formal, written annual budget^b^	1.25 (0.49–3.14)	1.42 (0.49–3.14)
Total congregational budget for most recent fiscal year		
Less than $250 K	Ref	Ref
Greater than $250 K	1.06 (0.49–2.32)	1.03 (0.12–9.01)
Follows the prosperity gospel^b^	2.58 (0.99–6.71)**	1.57 (0.29–8.38)
*Sociopolitical Characteristics*		
Groups, meetings, classes, or events to…		
Discuss politics^b^	0.59 (0.14–2.51)	1.30 (0.16–10.70)
Get people registered to vote^b^	5.36 (1.50–19.16)**	6.13 (0.28–133.20)
Discuss science & religion^b^	3.16 (1.23–8.15)**	1.64 (0.26–10.34)
Discuss race relations^b^	1.84 (0.74–4.54)	4.13 (0.81–20.99)*
Go vote during an election^b^	0.42 (0.14–1.24)	1.26 (0.10–15.98)
Discuss race & police^b^	2.92 (0.95–8.95)*	1.36 (0.12–15.82)
Discuss issues related to sexual orientation & gender identity^b^	2.78 (1.14–6.75)**	3.39 (0.52–22.15)
Lobbying or marching activities related to abortion^b^	4.41 (1.17–16.61)**	6.54 (0.28–151.27)
Lobbying or marching activities related to issues concerning gay, lesbian, or transgender people^b^	0.82 (0.16–4.25)	0.01 (0.00–3.29)

OR = odds ratio; CI = confidence interval. ***p < 0.001; **p < 0.05; *p < 0.10

^a^Data are from the 2018–2019 National Congregations Study

^b^0 = no, 1 = yes

### Sociodemographic Factors and Mental/Behavioral Health Programming

Table 1 presents odds ratios of sociodemographic factors associated with mental and behavioral health programming and/or support in U.S. congregations. Urban and suburban congregations with more than 500 attendees were more likely to offer such programming and/or support than urban and suburban congregations with fewer than 500 attendees (OR = 3.43, *p* < 0.01). Rural congregations that follow the prosperity gospel were more likely to offer such programming and/or support than rural congregations that did not follow the prosperity gospel (OR = 4.37, *p* < 0.05). Urban and suburban congregations that follow the prosperity gospel were more likely to offer such health programming and/or support than urban and suburban congregations that did not follow the prosperity gospel (OR = 2.61, *p* < 0.05).

### Sociodemographic and Sociopolitical Factors and Mental/Behavioral Health Programming

Table 2 presents odds ratios for offering mental health and behavioral programming and/or support among U.S. congregations, adjusting for sociodemographic and sociopolitical factors. Among sociodemographic characteristics, urban and suburban congregations with more than 500 attendees were more likely to offer programming and/or support than urban and suburban congregations with less than 500 attendees (OR = 3.36, *p* < 0.05). Urban and suburban congregations founded between 2000 and 2018 were less likely to offer health programming and/or support than urban and suburban congregations founded before 1900 (OR = 0.26, *p* < 0.05).

Among sociopolitical characteristics, urban and suburban congregations that held groups, meetings, classes, or events to get people registered to vote were more likely to offer mental and behavioral health programming and/or support than urban and suburban congregations that did not hold groups, meetings, classes, or events to get people registered to vote (OR = 5.36, *p* < 0.01). Urban and suburban congregations that held groups, meetings, classes, or events to discuss science and religion were more likely to offer mental and behavioral health programming and/or support than urban and suburban congregations that did not hold groups, meetings, classes, or events to discuss science and religion (OR = 3.16, *p* < 0.05).

Urban and suburban congregations that held groups, meetings, classes, or events to discuss issues related to sexual orientation and gender identity were more likely to offer mental and behavioral health programming and/or support than urban and suburban congregations that did not hold groups, meetings, classes, or events to discuss issues related to sexual orientation and gender identity (OR = 2.78, *p* < 0.05). Urban and suburban congregations that lobbied or marched for activities related to abortion were more likely to offer such health programming and/or support than urban and suburban congregations that did not lobby or march for activities related to abortion (OR = 4.41, *p* < 0.05). There were no statistically significant findings for rural congregations after adjusting for these sociodemographic and sociopolitical characteristics.

## Discussion

This study found that urban and suburban congregations were more likely to offer mental and behavioral health programs than rural congregations. This finding, as expected, shows how greater access to mental health resources in urban and suburban areas likely facilitates the ability of congregations in these settings to offer such programming. Rural congregations may be less likely to offer such programming to their attendees given limited resources. Additionally, rural congregations may be face challenges providing programming due to higher rates of mental health stigma and substance use in these areas (Meit et al., 2022).

Some attendees may be uncomfortable discussing their mental health in a public setting like a faith-based institution. For example, privacy and confidentiality may be a greater concern in rural congregations, which are typically smaller than urban and suburban congregations. The significant percentage of mental and behavioral health programming offered in rural congregations highlights how faith-based institutions are addressing a critical need in areas with limited access to such services (Mama et al., 2020; Voss, 1996). Many rural residents may prefer seeking mental health support from faith-based institutions over traditional providers due to the trusted role faith-based institutions hold in these areas (SAMHSA, 2023).

Among congregations offering mental and behavioral health programming, we found differences in sociopolitical factors amongst congregations in urban and suburban versus rural areas. Congregations with more than 500 attendees in urban and suburban areas were more likely than smaller congregations to provide programming. This is unsurprising as larger congregations may have more resources than smaller congregations. Congregations with female clergy were also more likely to offer programming, which may reflect gender differences in mental health stigma and perceptions among men (Chatmon, 2020).

In terms of sociopolitical leanings, urban and suburban congregations holding groups, meetings, classes, or events to get people registered to vote, as well as those engaging in similar activities around discussing science, religion, sexual orientation, and gender identity were more likely to offer mental and behavioral health programming than congregations not participating in such activities. Urban and suburban congregations lobbying or marching for activities related to abortion were more likely to provide such programming than congregations that do not, but the same associations were not found for rural congregations. Urban and suburban areas provide more opportunities for these topic discussions than rural areas.

Engagement in discussions and activities about sociopolitical issues may indicate that congregations are more receptive to providing mental and behavioral health support, which is often understood as a socially embedded issue (Kirkbride et al., 2024; Puttman & Burrows, 2024). Despite these differences, there are similarities in factors that may contribute to urban and suburban and rural congregations offering these programs. Congregations from both geographical areas with a fiscal budget higher than $250 K were more likely to offer mental and behavioral health programs. This is not surprising given that a higher budget likely means more financial resources to offer such programs.

Interestingly, congregations from both geographical areas following the prosperity gospel were more likely to offer mental and behavioral health programs. Given the prosperity gospel often promotes the belief that greater faith and financial contributions to the church can lead to relief from conditions such as depression and anxiety and result in improved health (Bowler, 2018; Gbote & Kgatla, 2014), further research is needed to assess the quality and nature of mental health resources offered in congregations that adhere to this theological framework. Our findings should raise some concern about the quality and evidence-based programming offered to congregants in those settings.

Many people may seek mental and behavioral health support from religious institutions, because they cannot access or afford other types of care or because they are more comfortable seeking support at their congregation. Given this observation and the high percentage of congregations identified in the survey as offering mental and behavioral health support or programming, it is important to further examine the nature, scope, and context in which these services are being provided. All support is not created equally and without sound evidence-based practices and appropriate referral systems, faith-based mental and behavioral health programming may fail to meet individuals'needs and, in some cases, may cause harm (Longo et al., 2013; Schieman et al., 2013). When effectively implemented, programming within faith-based institutions may address a critical service gap, particularly in rural areas.

### Limitations

This study has several limitations, including using data collected before the COVID-19 pandemic. Given the significant impact of the pandemic on mental health outcomes and access to services, this may have prompted more congregations both in rural and urban and suburban areas to provide mental health support and programming in the years since the start of the pandemic. While this study highlights factors associated with congregations’ likelihood of offering mental and behavioral health programming and supports, these data do not capture information regarding utilization of services or programming quality performance.

## Conclusion

Faith-based institutions are often trusted places in communities where people may feel comfortable receiving mental health support. These institutions often fill gaps, particularly in rural areas, where there is a shortage of formal mental health services. This study examines specific factors associated with offering mental and behavioral health programming in rural, urban and suburban congregations. Our findings show that while urban and suburban congregations are more likely to offer mental and behavioral health programs than rural congregations, there are still many complex sociodemographic, sociopolitical, and health-related characteristics of rural, urban and suburban congregations that impact their likelihood of offering such programs. Further research is needed to examine the quality of these programs, as well as the utilization of these programs by both rural, urban and suburban residents.

## Supplementary Information

Below is the link to the electronic supplementary material.

Supplementary file1 (DOCX 38 kb)
