# Supplementary material for: Factors Associated with Mental and Behavioral Health Programming in U.S. Rural, Urban and Suburban Congregations
Source: J Relig Health. 2025 Aug 9;64(6):4891–907. doi: 10.1007/s10943-025-02403-6 (PMC12518456; doi:10.1007/s10943-025-02403-6)
Supplement: Supplementary file 1 — Supplementary file1 (DOCX 38 kb) [file 10943_2025_2403_MOESM1_ESM.docx]

**Appendix**

**Appendix 1**

| *Sociodemographic, Sociopolitical, and Health-related Characteristics Among U.S. Rural, Urban and Suburban Congregations ^a^* | | | | |
| --- | --- | --- | --- | --- |
|  | Rural, Urban and Suburban Congregations | | | |
|  | Urban/Suburban | Rural | Total | *p* |
| *N (%)* | 950 (75.3%) | 312 (24.7%) | 1,262 (100%) |  |
| Sociodemographic Characteristics | | | | |
| Clergy race/ethnicity |  |  |  | 0.07 |
| White | 541 (57.0%) | 228 (73.0%) | 769 (60.9%) |  |
| Black or African American | 244 (25.6%) | 65 (20.9%) | 309 (24.5%) |  |
| Hispanic | 58 (6.1%) | 4 (1.4%) | 62 (4.9%) |  |
| Asian or Pacific Islander | 43 (4.5%) | 7 (2.2%) | 50 (4.0%) |  |
| Other | 1 (0.1%) | 0 (0.0%) | 1 (0.1%) |  |
| Unknown | 64 (6.7%) | 8 (2.4%) | 71 (5.7%) |  |
| Clergy sex |  |  |  | 0.12 |
| Male | 780 (82.1%) | 251 (80.4%) | 1,030 (81.6%) |  |
| Female | 107 (11.3%) | 54 (17.2%) | 161 (12.8%) |  |
| Unknown | 63 (6.6%) | 8 (2.4%) | 71 (5.6%) |  |
| Number of adult congregants |  |  |  | <0.01 |
| Less than 500 | 902 (94.9%) | 310 (99.4%) | 1,212 (96.0%) |  |
| Greater than 500 | 48 (5.1%) | 2 (0.6%) | 50 (4.0%) |  |
| Percent of female congregants |  |  |  | 0.79 |
| <25% | 15 (1.6%) | 2 (0.7%) | 17 (1.4%) |  |
| 25% - 49% | 58 (6.1%) | 17 (5.3%) | 74 (5.9%) |  |
| 50% - 74% | 740 (78.3%) | 237 (76.4%) | 976 (77.8%) |  |
| 75% - 100% | 132 (14.0%) | 54 (17.6%) | 186 (14.9%) |  |
| Percentage of congregants with a bachelor’s degree |  |  |  | <0.01 |
| <25% | 274 (31.9%) | 168 (63.4%) | 442 (39.4%) |  |
| 25% - 49% | 194 (22.6%) | 49 (18.7%) | 243 (21.7%) |  |
| 50% - 74% | 197 (22.9%) | 25 (9.4%) | 221 (19.7%) |  |
| 75% - 100% | 194 (22.6%) | 22 (8.5%) | 217 (19.3%) |  |
| Year congregation was officially founded |  |  |  | <0.01 |
| Before 1900 | 148 (16.0%) | 99 (32.9%) | 247 (20.1%) |  |
| 1900-1949 | 167 (18.0%) | 76 (25.2%) | 243 (19.7%) |  |
| 1950-1999 | 391 (42.1%) | 96 (31.8%) | 487 (39.6%) |  |
| 2000-2018 | 223 (24.0%) | 31 (10.1%) | 253 (20.6%) |  |
| Building ownership |  |  |  | <0.05 |
| Belongs to congregation or denomination | 771 (81.1%) | 291 (93.4%) | 1,062 (84.2%) |  |
| Belongs to another group | 179 (18.9%) | 21 (6.6%) | 200 (15.8%) |  |
| Type of building used for worship |  |  |  | 0.16 |
| School | 16 (1.7%) | 0 (0.0%) | 16 (1.3%) |  |
| Storefront | 40 (4.2%) | 0 (0.0%) | 40 (3.1%) |  |
| Church, synagogue, temple or mosque | 825 (86.8%) | 302 (97.0%) | 1,127 (89.3%) |  |
| Other | 70 (7.4%) | 9 (3.0%) | 79 (6.3%) |  |
| Region |  |  |  | <0.01 |
| Northeastern | 145 (15.2%) | 20 (6.4%) | 165 (13.1%) |  |
| Midwestern | 220 (23.2%) | 80 (25.8%) | 301 (23.8%) |  |
| Southern | 389 (41.0%) | 196 (62.8%) | 585 (46.4%) |  |
| Western | 196 (20.6%) | 16 (5.0%) | 211 (16.8%) |  |
| Percent White congregants |  |  |  | <0.01 |
| <25% | 361 (38.4%) | 79 (25.5%) | 440 (35.2%) |  |
| 25% - 49% | 33 (3.5%) | 0 (0.1%) | 33 (2.7%) |  |
| 50% - 74% | 77 (8.2%) | 7 (2.3%) | 84 (6.7%) |  |
| 75% - 100% | 470 (50.0%) | 223 (72.2%) | 693 (55.4%) |  |
| Percent Black congregants |  |  |  | 0.06 |
| <25% | 676 (72.0%) | 240 (77.9%) | 917 (73.4%) |  |
| 25% - 49% | 37 (3.9%) | 1 (0.3%) | 38 (3.0%) |  |
| 50% - 74% | 19 (2.0%) | 0 (0.0%) | 19 (1.5%) |  |
| 75% - 100% | 208 (22.1%) | 67 (21.8%) | 275 (22.0%) |  |
| Percent Latino congregants |  |  |  | 0.05 |
| <25% | 834 (88.6%) | 299 (97.0%) | 1,133 (90.7%) |  |
| 25% - 49% | 37 (4.0%) | 2 (0.8%) | 40 (3.2%) |  |
| 50% - 74% | 10 (1.1%) | 2 (0.8%) | 12 (1.0%) |  |
| 75% - 100% | 60 (6.4%) | 5 (1.5%) | 64 (5.2%) |  |
| Percent Asian congregants |  |  |  | 0.08 |
| <25% | 874 (93.0%) | 308 (99.8%) | 1,182 (94.7%) |  |
| 25% - 49% | 17 (1.8%) | 0 (0.0%) | 17 (1.3%) |  |
| 50% - 74% | 9 (0.9%) | 0 (0.0%) | 9 (0.7%) |  |
| 75% - 100% | 41 (4.3%) | 0 (0.2%) | 41 (3.3%) |  |
| Percent Other congregants |  |  |  | 0.58 |
| <25% | 901 (96.2%) | 302 (97.8%) | 1,203 (96.6%) |  |
| 25% - 49% | 2 (0.2%) | 2 (0.7%) | 4 (0.3%) |  |
| 50% - 74% | 15 (1.6%) | 0 (0.0%) | 15 (1.2%) |  |
| 75% - 100% | 18 (1.9%) | 5 (1.5%) | 23 (1.8%) |  |
| 80% of congregation are Black/African American ^b^ | 47 (5.0%) | 1 (0.4%) | 49 (3.9%) | <0.05 |
| 30% of congregation are below federal poverty line ^b^ | 167 (17.6%) | 26 (8.3%) | 193 (15.3%) | 0.02 |
| Congregation affiliated with a denomination convention or other kind of association ^b^ | 787 (82.8%) | 249 (79.8%) | 1,035 (82.0%) | 0.54 |
| Congregation has a website on the internet ^b^ | 746 (78.8%) | 151 (49.4%) | 897 (71.6%) | <0.01 |
| Congregation has a Facebook page ^b^ | 723 (76.4%) | 188 (60.7%) | 911 (72.5%) | <0.05 |
| Operates with a formal, written annual budget ^b^ | 710 (76.0%) | 230 (75.5%) | 940 (75.9%) | 0.94 |
| Total congregational budget for most recent fiscal year |  |  |  | <0.05 |
| Less than $20k | 67 (9.7%) | 28 (12.4%) | 95 (10.4%) |  |
| $20k - $49,999 | 108 (15.5%) | 43 (19.0%) | 151 (16.4%) |  |
| $50k - $249,999 | 309 (44.6%) | 133 (58.4%) | 442 (48.0%) |  |
| $250k - $999,999 | 154 (22.2%) | 19 (8.3%) | 172 (18.7%) |  |
| Greater than 1,000,000+ | 55 (8.0%) | 4 (2.0%) | 60 (6.5%) |  |
| Follows the prosperity gospel ^b^ | 190 (20.0%) | 86 (27.6%) | 277 (21.9%) | 0.18 |
| Sociopolitical Characteristics | | | | |
| Groups, meetings, classes, or events to... |  |  |  |  |
| Discuss politics ^b^ | 96 (10.1%) | 37 (11.9%) | 133 (10.5%) | 0.67 |
| Get people registered to vote ^b^ | 223 (23.5%) | 64 (20.6%) | 287 (22.8%) | 0.63 |
| Discuss science & religion ^b^ | 146 (15.3%) | 37 (12.0%) | 183 (14.5%) | 0.35 |
| Discuss race relations ^b^ | 310 (32.7%) | 52 (16.6%) | 362 (28.7%) | <0.05 |
| Go vote during an election ^b^ | 246 (25.8%) | 87 (27.8%) | 332 (26.3%) | 0.74 |
| Discuss race & police ^b^ | 199 (21.0%) | 37 (11.8%) | 236 (18.7%) | 0.09 |
| Discuss issues related to sexual orientation & gender identity ^b^ | 205 (21.6%) | 38 (12.3%) | 243 (19.3%) | <0.05 |
| Allows a same sex wedding to take place in their building ^b^ | 139 (14.6%) | 27 (8.5%) | 165 (13.1%) | 0.27 |
| Lobbying or marching activities related to abortion ^b^ | 62 (6.6%) | 10 (3.1%) | 72 (5.7%) | 0.08 |
| Pro-life side or pro-choice position |  |  |  | 0.10 |
| Pro-choice | 7 (0.7%) | 4 (1.3%) | 11 (0.9%) |  |
| Pro-life | 51 (5.4%) | 6 (1.8%) | 57 (4.5%) |  |
| No answer | 892 (93.9%) | 302 (96.9%) | 1,194 (94.6%) |  |
| Lobbying or marching issues related to LGBT people ^b^ | 44 (4.7%) | 4 (1.3%) | 48 (3.8%) | 0.08 |
| Health-related Programming | | | | |
| Programs involving substance abuse or addiction ^b^ | 31 (3.3%) | 5 (1.7%) | 37 (2.9%) | 0.31 |
| Programs involving health education ^b^ | 20 (2.1%) | 4 (1.4%) | 25 (2.0%) | 0.69 |
| Programs involving physical fitness ^b^ | 12 (1.2%) | 0 (0.1%) | 12 (0.9%) | <0.01 |
| Programs focusing on physical health needs ^b^ | 184 (19.4%) | 41 (13.3%) | 226 (17.9%) | 0.17 |
| Support to persons living with HIV or AIDS ^b^ | 158 (16.6%) | 16 (5.2%) | 174 (13.8%) | <0.01 |
| Programs to exercise or promote physical activity ^b^ | 389 (40.9%) | 66 (21.2%) | 455 (36.0%) | <0.01 |
| Programs involving mental health ^b^ | 6 (0.6%) | 0 (0.0%) | 6 (0.5%) | 0.14 |
| Programs targeting substance abuse ^b^ | 8 (0.8%) | 7 (2.4%) | 15 (1.2%) | 0.32 |
| Support for people with mental illness ^b^ | 266 (28.0%) | 63 (20.3%) | 329 (26.1%) | 0.20 |
| Support for people struggling with drug or alcohol abuse ^b^ | 433 (45.6%) | 105 (33.6%) | 538 (42.6%) | 0.05 |
| *Note*: mean (standard deviation): p-value from linear regression. Frequency (percent%): p-value from Pearson test. This table displays weighted statistics.  ^a^ Data are from the 2018‐2019 National Congregations Study.  ^b^ 0 = no, 1 = yes. | | | | |

**Appendix 2**

| *Mental/Behavioral Health Programming and/or Support Among U.S. Rural, Urban and Suburban Congregations ^a^* | | | | |
| --- | --- | --- | --- | --- |
| Mental/Behavioral Health Programming and/or Support | Rural, Urban and Suburban Congregations | | | |
|  | Urban/Suburban | Rural | Total | *p* |
| *N (%)* | 950 (75.3%) | 312 (24.7%) | 1,262 (100.0%) |  |
| Provided programs/support for people with mental illness |  |  |  | 0.13 |
| No programs and/or support | 456 (48.0%) | 180 (57.7%) | 636 (50.4%) |  |
| Any programs and/or support | 494 (52.0%) | 132 (42.3%) | 626 (49.6%) |  |
| Provided programs/support for people struggling with drug or alcohol abuse |  |  |  | 0.12 |
| No programs and/or support | 517 (54.4%) | 200 (64.1%) | 717 (56.8%) |  |
| Any programs and/or support | 433 (45.6%) | 112 (35.9%) | 545 (43.2%) |  |
| Only provided programs that involved mental health conditions |  |  |  | 0.18 |
| No programs | 681 (71.6%) | 248 (79.7%) | 929 (73.6%) |  |
| Any programs | 270 (28.4%) | 63 (20.3%) | 333 (26.4%) |  |
| *Note*: mean (standard deviation): p-value from linear regression. Frequency (percent%): p-value from Pearson test. This table displays weighted statistics.  *^a^* Data are from the 2018‐2019 National Congregations Study. | | | | |
